# Selection processes in simple sequence repeats suggest a correlation with their genomic location: insights from a fungal model system

**DOI:** 10.1186/s12864-015-2274-x

**Published:** 2015-12-29

**Authors:** Paolo Gonthier, Fabiano Sillo, Elisa Lagostina, Angela Roccotelli, Olga Santa Cacciola, Jan Stenlid, Matteo Garbelotto

**Affiliations:** Department of Agricultural, Forest and Food Sciences, University of Torino, 10095 Grugliasco, Italy; Department of Environmental Sciences, Policy and Management, University of California at Berkeley, CA 94720 Berkeley, USA; Department of Earth and Environmental Sciences, University of Pavia, 27100 Pavia, Italy; Department of Agriculture, Mediterranean University of Reggio Calabria, 89122 Reggio Calabria, Italy; Department of Agriculture, Food and Environment, University of Catania, 95123 Catania, Italy; Department of Forest Mycology and Plant Pathology, Swedish University of Agricultural Sciences, 75007 Uppsala, Sweden

**Keywords:** Adaptive evolution, *Heterobasidion*, Population genetics, SSRs

## Abstract

**Background:**

Adaptive processes shape the evolution of genomes and the diverse functions of different genomic regions are likely to have an impact on the trajectory and outcome of this evolution. The main underlying hypothesis of this study is that the evolution of Simple Sequence Repeats (SSRs) is correlated with the evolution of the genomic region in which they are located, resulting in differences of motif size, number of repeats, and levels of polymorphisms. These differences should be clearly detectable when analyzing the frequency and type of SSRs within the genome of a species, when studying populations within a species, and when comparing closely related sister taxa. By coupling a genome-wide SSR survey in the genome of the plant pathogenic fungus *Heterobasidion irregulare* with an analysis of intra- and interspecific variability of 39 SSR markers in five populations of the two sibling species *H. irregulare* and *H. annosum*, we investigated mechanisms of evolution of SSRs.

**Results:**

Results showed a clear dominance of trirepeats and a selection against other repeat number, i.e. di- and tetranucleotides, both in regions inside Open Reading Frames (ORFs) and upstream 5’ untranslated region (5’UTR). Locus per locus AMOVA showed SSRs both inside ORFs and upstream 5’UTR were more conserved within species compared to SSRs in other genomic regions, suggesting their evolution is constrained by the functions of the regions they are in. Principal coordinates analysis (PCoA) indicated that even if SSRs inside ORFs were less polymorphic than those in intergenic regions, they were more powerful in differentiating species. These findings indicate SSRs evolution undergoes a directional selection pressure comparable to that of the ORFs they interrupt and to that of regions involved in regulatory functions.

**Conclusions:**

Our work linked the variation and the type of SSRs with regions upstream 5’UTR, putatively harbouring regulatory elements, and shows that the evolution of SSRs might be affected by their location in the genome. Additionally, this study provides a first glimpse on a possible molecular basis for fast adaptation to the environment mediated by SSRs.

**Electronic supplementary material:**

The online version of this article (doi:10.1186/s12864-015-2274-x) contains supplementary material, which is available to authorized users.

## Background

Microsatellites or Simple Sequence Repeats (SSRs), defined as loci comprising tandem repeats of 1 (referred to as homopolymeric tracts) to 6 base pairs, are ubiquitous both in prokaryotic and eukaryotic genomes [1]. Historically described as “junk” DNA, the role of SSRs in evolution has been re-evaluated by recent findings thanks to the great expansion of genomic research [2]. These peculiar sequences show high rate of reversible mutation in length, ranging from 10^−7^ to 10^−3^ number of mutations per generation, which is higher than the average genomic rate of point mutations normally evaluated at 10^−8^–10^−9^ [3]. Mutation rate can be affected by motif length, motif sequence, number of repeats, and imperfection of SSRs (e.g., imperfect repeats are more stable than perfect repeats) [3]. It has been demonstrated that SSRs are distributed non-randomly in genomes, probably because of their varied effects on functions including chromatin organization, gene activity regulation, DNA replication and recombination, cell cycle, and mismatch repair system [4]. Within eukaryotes, each species may have a distinct motif frequency distribution [2].

For all of the abovementioned reasons, SSRs are powerful and widely used genetic markers. Their co-dominant nature and high levels of polymorphisms make them invaluable for genotyping purposes as well as for standard population genetic analyses. Their presumed stepwise evolution pattern can also be utilized to study how diversity is generated when invasive populations are started from one or a few founder genotypes, as it has been done for the pathogen causing the Sudden Oak Death epidemic [5]. Moreover, some SSRs are hypervariable, allowing studies of evolution and genetic structure of clonal species or populations. While several approaches have been extensively used to isolate SSR loci [6], the availability of an annotated genome is the most powerful approach to identify SSRs and to study their distribution, type, and evolution. Presence and extent of SSRs variability is likely to be affected by several factors including reproductive behaviour, effective size and age of populations, and other intrinsic factors linked to DNA replication and transcription. It has been postulated that SSRs may have a role in modulating gene expression [7]: the type and frequency of SSRs in regions known to be responsible for gene regulation may corroborate such a role, and may also allow for the better understanding of general genomic patterns of evolution and variability.

Even though SSR loci have been extensively described as part of the annotation effort of several fungal genomes, their roles in genome shaping, and their contribution to genome evolution, are still unclear. Recent studies showed that they are not randomly distributed throughout the genomes and they seem not to always follow the classical stepwise mutation based on neutral or nearly neutral theory [8].

SSRs are less frequent in fungal than in other eukaryotic genomes, although each species appears to have a unique signature of repeat distribution across the Kingdom Fungi [9]. Interestingly, genome size seems not to be correlated with SSR density [7, 9]. In the yeast *Saccharomyces cerevisiae* Meyen ex E.C. Hansen, it has been reported that about 75 % of predicted gene models harbouring SSRs encoded for proteins related to cell surface involved in cell adhesion and flocculation [10]. Cumulatively, these findings suggest that in addition to contributing in genome shaping, SSRs could play active role during adaptive evolutionary processes [11, 12].

In the current work, we used the two sibling plant pathogenic fungal species *Heterobasidion irregulare* Otrosina & Garbel. and *H. annosum* (Fr.) Bref. as a model system to assess how selection drives SSRs type and variability during divergent evolution processes. These species are regarded as two of the most destructive forest pathogens in North America and Eurasia, respectively [13]. Both phylogenetic and comparative genomics studies indicated that *H. irregulare* and *H. annosum,* which have retained high level of interfertility [14]*,* are monophyletic sister taxa which have diverged genetically approximately 34–41 millions of years (MY) ago and evolved into distinct allopatric species [15–17]. The definitive evidence on their status as distinct species was obtained recently through a phylogenomic approach comparing several *H. irregulare* and *H. annosum* genotypes [18]. Due to the accidental introduction of *H. irregulare* into Italy during World War II, the two species have attained sympatry [19] and started admixing their genomes [20, 21]. Outside of the area of sympatry, currently limited to the Latium region of Italy [22, 23], individuals belonging to the two species have not exchanged genes for at least 34–41 MY [17], thus allowing for a rigorous comparative analysis.

The general aims of this study were: A- to confirm that the distribution of SSRs is not random in terms of frequency of each SSR type within the fully annotated genome of *H. irregulare*; and, B- to corroborate the evolutionary assumptions of a non-random (or random) distribution of SSR, by testing whether the genomic location of trimers is correlated to traits such as levels of polymorphism and homoplasy. In order to do so, a whole genome survey of SSRs in the *H. irregulare* genome was carried out to categorize type and location of all SSRs and to identify specific trinucleotidic SSR markers in clearly distinct genomic regions. This survey allowed us to identify SSRs located within Open Reading Frames (ORFs) or clearly upstream and downstream of 5’ and 3’ untranslated regions (UTRs), respectively. Coupling the SSR survey with a population genetic analysis, our specific goals were: I) to determine the distribution and identify the motifs of the different SSR categories in selected genomic regions of the *H. irregulare* genome, i.e. inside ORFs, inside UTRs, immediately upstream and downstream of UTRs, as well as far from transcribed regions and to compare patterns with that of other fungal genomes; II) to compare the amount of intraspecific and interspecific variability of trinucleotide SSRs located in the abovementioned genomic regions; and III) to test if SSRs in different regions may evolve in concert with the region they are inserted in, resulting in loci with different analytical phylogenetic resolution due to different levels of homoplasy or constraints on number of repeats [7].

## Results

### Distribution of SSRs in the *H. irregulare* genome

**Fig. 1:**
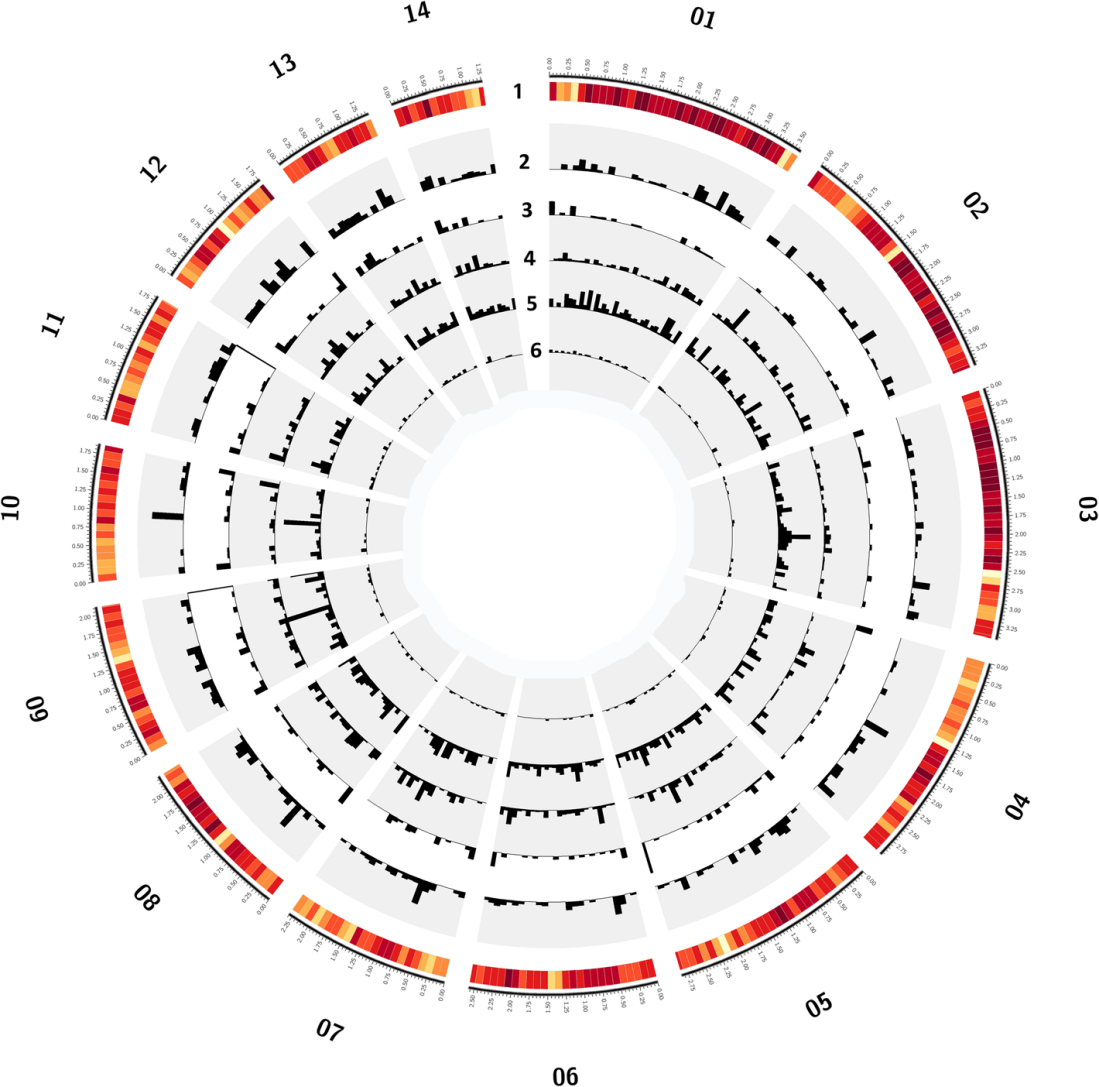
SSRs distribution across different scaffolds (chromosomes) of the *H. irregulare* reference genome. The 14 scaffolds corresponding to chromosomes are shown. Gene density (1) in a window size of 20 Kbp is colour coded from light yellow to dark red, with deeper red region representing high gene density. From outer to inner, black histograms represent distribution of hexa- (track 2), penta- (track 3), tetra- (track 4), tri- (track 5), di- (track 6) nucleotidic SSRs, respectively. Histogram size represent frequency of SSRs in a window size of 10 Kbp. Figures were drawn by using Circos software v. 0.64 (http://circos.ca/)

Based on our search parameters, the total number of perfect SSRs was 2541, representing about 0.0017 % of the *H. irregulare* genome. There was approximately one microsatellite *per* 13.26 Kbp and the total numbers of SSRs in intergenic and intragenic regions were similar, 1372 *versus* 1169 (Fig. 1).

Chi-square test indicated that the distribution of SSRs was not random (*p*-value < 0.05) throughout the genome. Frequency of SSRs *per* Mbp in the ten largest scaffolds was highest in 3’UTR, followed by regions located more than 500 bp from UTRs, regions 50 bp upstream 5’UTR (presumably spanning into the promoter region), 5’UTR, 50 bp downstream 3’UTR, 50–500 bp downstream 3’UTR, introns, 50–500 bp upstream 5’UTR and exons.

**Fig. 2:**
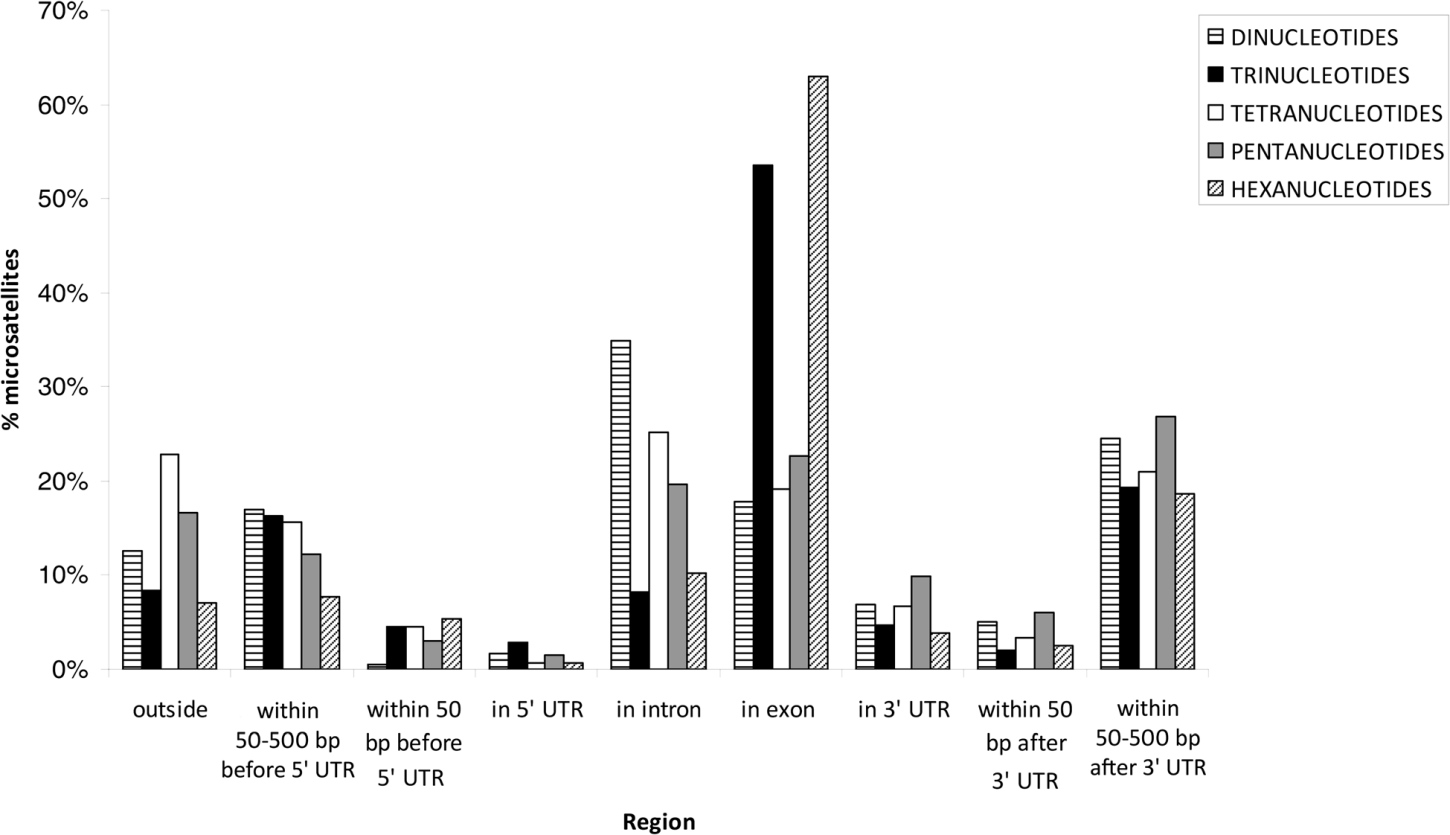
Frequency of different types of SSRs in selected genomic regions of the *H. irregulare* genome. Frequencies were estimated as the number of SSRs per Mbp

**Fig. 3:**
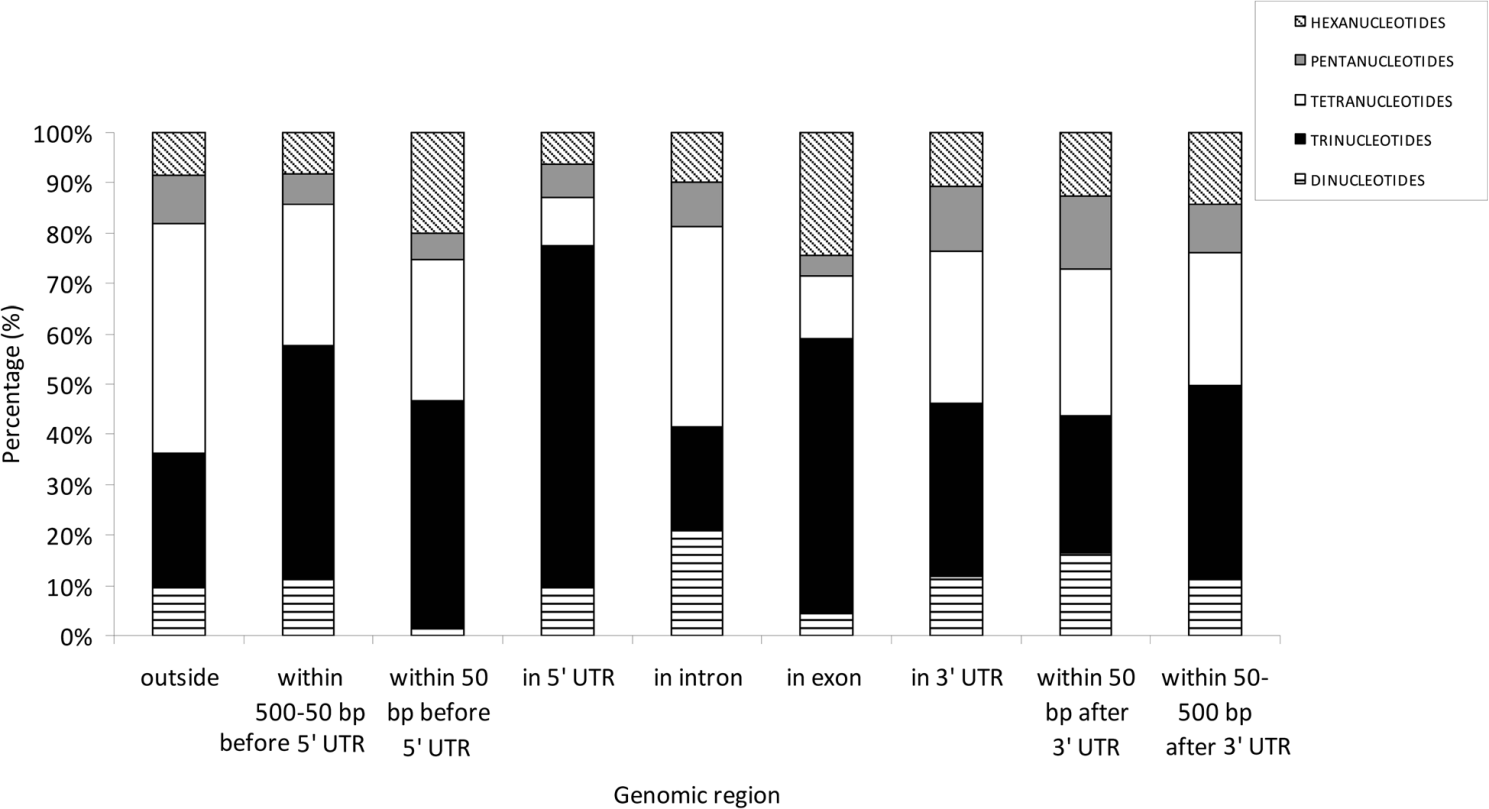
Percentage of different types of SSRs in selected genomic regions of the *H. irregulare* genome

The most frequent SSRs in exons were trinucleotides followed by hexanucleotides, while tetranucleotides were dominant in introns (Fig. 2). Trinucleotides were also clearly dominant in 5’UTRs, within 50 bp before 5’UTR and in 50–500 bp upstream 5’UTR. Conversely, tetranucleotides were more frequent than other SSRs in the genome fraction located more than 500 bp from transcribed regions. Frequencies of tri- and tetranucleotides are similar in 3’UTR and 50 bp downstream 3’UTR. Dinucleotides were present at extremely low frequencies both in exons and within 50 bp before 5’UTR. The frequency of SSRs was higher in 3’ than in 5’UTRs. Overall, the highest concentration of trinucleotides was found in 5’UTRs and within 50 bp before 5’UTR (Fig. 3).

**Fig. 4:**
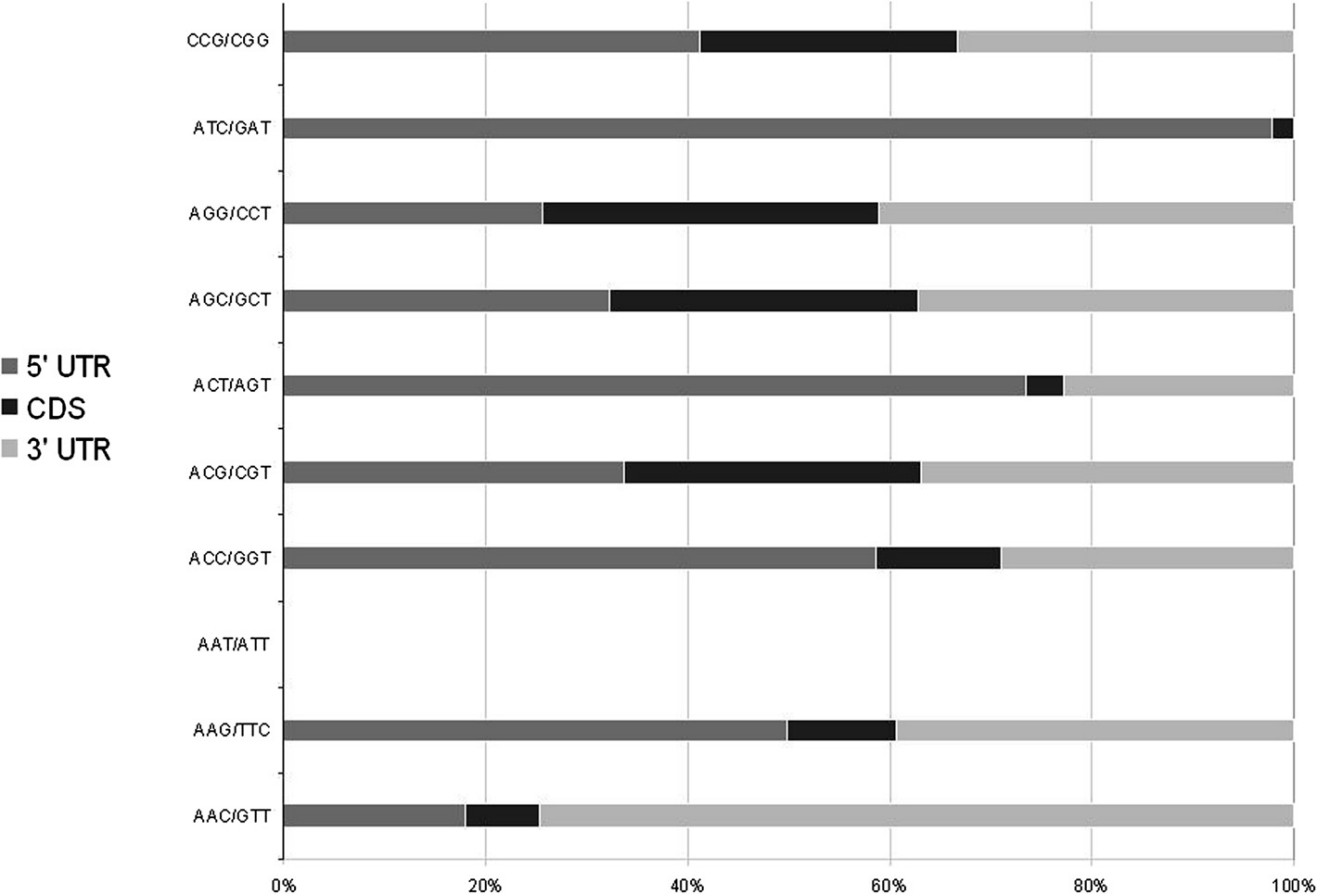
Percentages (%) of different trinucleotidic SSR motifs in transcribed regions. Coding sequences (CDSs) and UTRs were grey-scale coded

The most frequent perfect fully standardized motifs were ACG, CCG and AGC. Some repeats frequently reported in fungi (i.e. AT, ATC) [24] were rare in the *H. irregulare* genome (Table 1). On the other hand, *H. irregulare* harboured a significant component of repeats absent or rarely reported in fungi (i.e. CG, ACG) (Table 1). In transcribed regions, trinucleotidic motifs were surveyed and are summarized in Fig. 4.

**Table 1:** Genomic distribution of different trinucleotide repeats in *H. irregulare* and other fungi. Total length (bp per megabase of DNA) of fully standardized trinucleotide repeats in different genomic regions of the *H. irregulare* genome and of other fungal genomes was shown

Motif	*H. irregulare*						Fungi^a^			
	All	Inter^b^	Introns	Exons	5’UTR	3’UTR	All	Inter^b^	Introns	Exons
AAC	26	17	10	26	63	263	108	104	145	107
AAG	20	27	5	16	72	57	59	84	34	55
AAT	11	26	5	-	-	-	119	162	537	34
ACC	53	42	17	63	299	148	36	60	14	26
ACG	128	88	79	174	198	218	12	6	–	12
ACT	17	16	53	5	105	33	15	30	–	5
AGC	108	82	52	144	152	175	55	19	103	67
AGG	65	50	31	88	67	108	31	34	45	27
ATC	14	26	-	4	190	-	32	43	53	22
CCG	125	73	105	170	274	222	18	8	20	26

^a^ data from [24]

^b^ Intergenic region

Analysis of SSRs on coding sequences (CDSs) of filtered gene models showed that 766 CDSs contain at least one SSR. Fisher’s exact test on coding sequences containing SSRs *versus* all coding sequences of *H. irregulare* genome showed that GO terms GO:0055114 (oxidation-reduction process), GO:0006355 (regulation of transcription), GO:0009069 (serine family amino acid metabolic process) and GO:0005667 (transcription factor complex) were significantly over-represented (*p*-value < 0.05, False Discovery Rate - FDR < 0.05).

### SSRs amplification and polymorphism analysis

SSRs polymorphisms were scored by analyzing 39 SSR loci in different genomic regions, i.e. loci localized more than 500 bp upstream from transcribed regions (referred to as OUT), loci positioned within 500–50 bp before 5’UTR (UP), loci inside exons (IN) and loci within 50–500 bp after 3’UTR (DOWN). All SSR loci were polymorphic, showing 4 to 15 alleles per locus. Allelic sizes ranged from 82 to 674 and repeat number ranged from 2 to 191 (Additional file 1). The *H. irregulare* populations had a mean number of alleles of 6.636 ± 0.937 for OUT loci, 6.111 ± 0.389 for UP loci, 3.154 ± 0.373 for IN loci and 7.333 ± 1.054 for DOWN loci. The *H. annosum* populations had a mean number of alleles of 11.182 ± 1.166 for OUT loci, 11.222 ± 0.795 for UP loci, 5.538 ± 0.945 for IN loci and 11.500 ± 0.992 for DOWN loci (Table 2).

**Table 2:** Allelic diversity for the populations of *H. irregulare* and *H. annosum*. Number of different alleles, number of effective alleles and number of private alleles were shown. Loci localized more than 500 bp upstream from transcribed regions were referred to as OUT, loci positioned within 500–50 bp before 5’UTR were referred to as UP, loci inside exons were referred to as IN and loci within 50–500 bp after 3’UTR were referred to as DOWN

	*N*° of Different Alleles	*N*° of Effective Alleles (1 / (Sum pi^2^))	*N*° Private Alleles	*N*° of Shared Alleles
OUT				
*H. irregulare*	6.636 ± 0.937	3.900 ± 0.542	1.545 ± 0.666	5.091 ± 0.271
*H. annosum*	11.182 ± 1.166	5.485 ± 0.666	6.091 ± 0.847	5.091 ± 0.310
UP				
*H. irregulare*	6.111 ± 0.389	2.881 ± 0.223	1.333 ± 0.289	4.778 ± 0.100
*H. annosum*	11.222 ± 0.795	4.995 ± 0.520	6.444 ± 1.015	4.778 ± 0.495
IN				
*H. irregulare*	3.154 ± 0.373	2.124 ± 0.258	1.231 ± 0.303	1.923 ± 0.070
*H. annosum*	5.538 ± 0.945	2.907 ± 0.537	3.615 ± 0.665	1.923 ± 0.280
DOWN				
*H. irregulare*	7.333 ± 1.054	4.326 ± 0.559	1.000 ± 0.365	6.333 ± 0.689
*H. annosum*	11.500 ± 0.992	4.917 ± 0.621	5.167 ± 0.872	6.333 ± 0.120

### Population genetics analysis

**Fig. 5:**
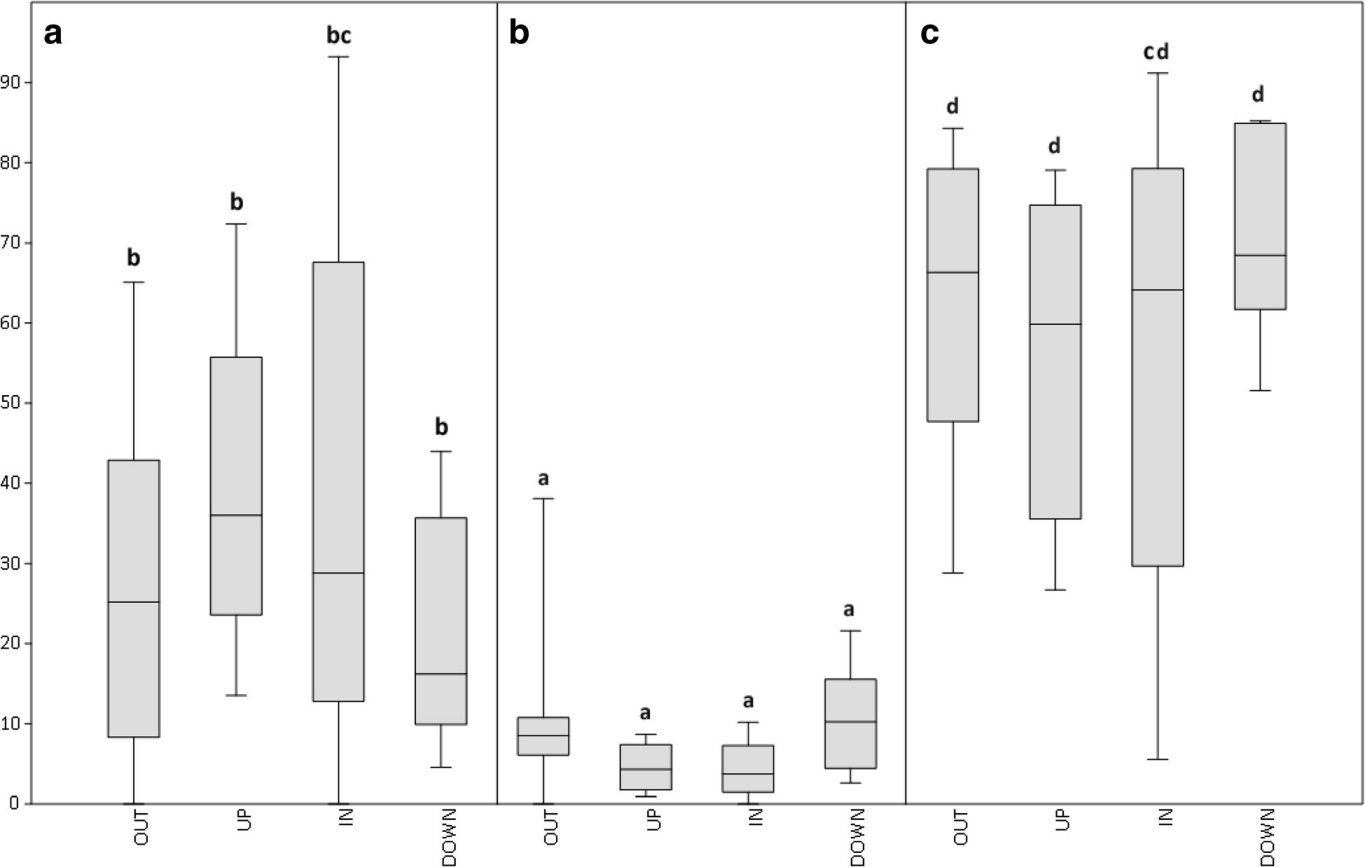
Boxplot representing variance components (%) *per* locus on populations of *H. irregulare* and *H. annosum*. **a** Variation between species; (**b**) Variation among populations; (**c**) Variation among genotypes. In the Y-axis the percentages of variation were plotted, while in X-axis the different categories of loci were represented. Boxplots with different letters are significantly different (F-test, *p*-value < 0.05)

**Fig. 6:**
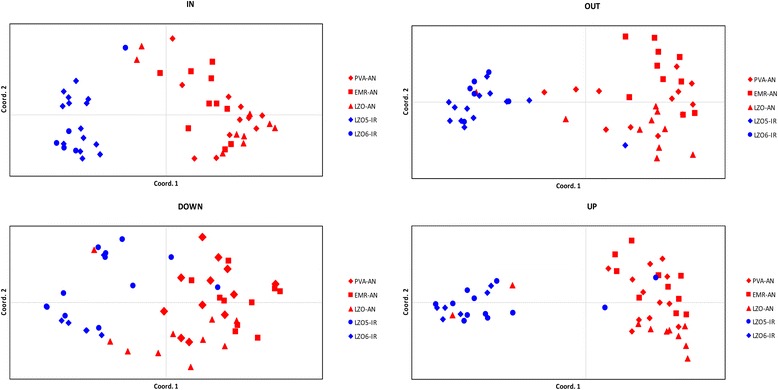
PCoA scatter plots showing the genetic distance among genotypes. Genotypes of *H. irregulare* (LZO5-IR, LZO6-IR) from 2 distinct sampling populations, both from Latium, and genotypes of *H. annosum* (PVA-AN, EMR-AN, LZO-AN) belonging to 3 distinct sampling populations from various regions of Northern and central Italy, including Latium, were considered

AMOVAs were performed on 51 *Heterobasidion* genotypes from two *H. irregulare* and three *H. annosum* populations. Results including all loci indicated that most of the genetic variability (54–71 %) was among genotypes within population, 3–9 % of the genetic variance was among populations within species, and 25–43 % of the molecular variance was between the two species (Table 3). Averaging all loci and populations, Fst values were as high as 0.40 (SD 0.22, *p*-value  < 0.05)*.* Average Fst values by each of the abovementioned categories were as follows: 0.37 for OUT loci, 0.42 for UP loci, 0.45 for IN loci, and 0.30 for DOWN loci. Details on percentage of *per* locus variance, as well as Fst and gene flow (2 Nm) values, calculated considering the genotypes as haploids, are summarized in Fig. 5 and Additional file 2, respectively. The genetic diversity detected with the 39 loci was sufficiently informative to reflect the genetic distinction between species. The PCoA showed a clear genetic differentiation between *H. irregulare* and *H. annosum*, especially when UP and IN loci were considered (Fig. 6). In IN loci no overlap between species was observed, while in OUT, UP, and DOWN loci partial overlap was observed between *H. irregulare* genotypes and *H. annosum* genotypes from the Latium population (LZO-AN) (Fig. 6)*.*

**Table 3:** Hieratical analysis of molecular variance (AMOVA). The genetic diversity between species, among populations and among genotypes within populations of 51 genotypes of *H. irregulare* and *H. annosum* was shown. Loci localized more than 500 bp upstream from transcribed regions were referred to as OUT, loci positioned within 500–50 bp before 5’UTR were referred to as UP, loci inside exons were referred to as IN and loci within 50–500 bp after 3’UTR were referred to as DOWN. Degrees of freedom (d.f.) were also included

Source of variation	d.f.	Sum of square	Variance components	percentage variation (%)
OUT				
Between species	1	71.086	1.27047	30.61
Among populations within species	3	29.382	0.37090	8.94
Among genotypes within populations	97	243.433	2.50962	60.46
Total	101	343.901	4.15099	100.00
UP				
Between species	1	62.024	1.22320	42.53
Among populations within species	3	10.512	0.09931	3.45
Among genotypes within populations	97	150.690	1.55350	54.02
Total	101	223.226	2.87602	100.00
IN				
Between species	1	25.901	0.51177	32.45
Among populations within species	3	7.127	0.07491	4.75
Among genotypes within populations	97	92.105	0.99038	62.80
Total	101	125.133	1.57706	100.00
DOWN				
Between species	1	22.562	0.41370	24.74
Among populations within species	3	8.064	0.07675	4.59
Among genotypes within populations	97	114.521	1.18062	70.65
Total	101	145.147	1.67107	100.00

Locus *per* locus AMOVA showed that for two OUT loci (MS38 and MS61), two IN loci (MS13 and MS19) and two DOWN loci (MS34 and MS69) the 2 Nm value was over 2 and the Fst value was less than 0.2 (Additional file 2). The IN locus MS13 showed the highest variability among genotypes within populations (91.16 %), while the OUT locus MS01 showed the highest variability among populations (38.10 %). The IN locus MS74 showed the highest value for variability between species (93.22 %) (Additional file 2). The percentages of variation between species resulting from the AMOVA varied depending on loci categories and were 30.61, 42.53, 32.45 and 24.74 for OUT, UP, IN and DOWN loci, respectively (Table 3).

## Discussion

### Distribution of SSRs in the sequenced *H. irregulare* genome

Although the frequency of SSRs *per *Mbp in *H. irregulare* was comparable between introns and exons, there was a clear and significant selection in favour of trinucleotides and hexanucleotides in exons *versus* introns. Such dominance of triplets over other repeats in coding regions may be explained on the basis of the suppression of nontrimeric SSRs in coding regions, i.e. dinucleotidic, tetranucleotidic and pentanucleotidic, possibly caused by lethal frame-shift mutations [7, 25–27]. It has been demonstrated that tri- and hexanucleotides repeats occur more frequently in coding regions than in non-coding regions of many eukaryotic genomes [9].

There is strong evidence that frequencies of different repeat motif of SSRs in coding regions vary depending on the type of the reciprocal encoded amino acid [7]. According to our GO analysis, in the *H. irregulare* genome there is an over-representation of SSRs in genes affecting transcription. By combining these findings, it could be argued that dominance of trirepeats, selection against other repeat numbers (in particular di- and tetrarepeats), presence of SSRs in genes related to transcriptional factors may be indicative of a role played by SSRs in gene regulation, for instance by altering the abundance of nuclear protein binding sites. It has been already reported in yeast that trinucleotides repeats seem to be common in genes encoding for transcriptional factors [28]. Variation in repeat length caused by extension or contraction of trinucleotides repeats may thus affect homopolymeric aminoacid sequences, leading to modifications of protein flexibility and/or binding affinity [2], as shown by changes in number of CCG repeats in humans [29]. The frequency of trinucleotides decreases further away from ORFs, and tetranucleotides dominate in regions further than 500 bp from UTRs. Interestingly, a similar pattern is observed in introns, where tetranucleotides are dominant compared to trinucleotides. This finding is surprising since this dominance in introns is reported only for vertebrates [30, 31]. Although intergenic regions and introns may be involved in the regulation of gene expression [12], such a pattern may suggest a loss of constraint that might be expected in regions less directly affected by changes in reading frame. Other fungal species analyzed at genomic level, including *Tuber melanosporum* and *Laccaria bicolor*, have showed strong constraint against tetranucleotidic SSRs in intronic regions [26, 32, 33].

Trinucleotides were dominant in the 5’UTRs and significantly less abundant than expected both in 3’UTRs and downstream of 3’UTRs. Dominance of trirepeats in the 5’UTR has been generally reported for both animals and plants [7], however, the *H. irregulare* genome is characterized by a higher frequency of SSRs in the 3’UTR, a trait commonly associated with animals and not plants [34, 35]. This trait also differentiates *H. irregulare* from the symbiont mushroom *Laccaria bicolor,* characterized by a notable absence of SSRs in 3’UTR regions [33]. The overall low number of SSRs in the *H. irregulare* genome, the clear dominance of trinucleotides in both the 5’UTR and within 50 bp upstream 5’UTR, and the low representation of dinucleotides within 50 bp upstream 5’UTRs suggest a history of negative selection towards those SSRs (dinucleotides) that are more likely to disrupt the functions played by these regions. In this scenario, dinucleotides might be harmful or costly and hence promptly purged by *H. irregulare*.

Results of the analysis on specific SSRs motifs were both expected and unexpected. The most abundant triplet found in introns was ACG, a motif reported as abundant in several Basidiomycetes species [36]. Unexpectedly, CG was also abundant, despite its reported underrepresentation in most organisms, including several fungi. Additionally, the AAT motif, reported as common in transcribed regions for many fungal species, was found to be absent in regions of *H. irregulare* with similar function [9].

### Intraspecific and interspecific variability of trinucleotide SSRs in different genomic regions

AMOVA results obtained from the analyses of the two *H. irregulare* populations and of the three *H. annosum* populations showed consistently a larger variability within populations than among populations, independent of location of the SSR locus. Greater variation was detected among genotypes within populations (ranging from 54 to 71 %) and between the two species (ranging from 25 to 43 %), than among populations of the same species (about 6 %). These results suggest a lack of genetically differentiated populations for both species in Italy. As expected, native *H. annosum* populations showed higher level of polymorphisms than the invasive *H. irregulare* populations, which have recently undergone the bottleneck of the introduction from North America into Italy [22].

Although SSRs within ORFs (IN) were less polymorphic than SSRs located elsewhere, they differentiated the two species as well as markers immediately upstream of 5’UTRs (UP). Additionally, both types of markers, IN and UP, were more powerful in differentiating the two species than loci clearly located away from transcribed regions (DOWN and OUT). Variability of SSRs immediately upstream 5’UTRs and within ORFs may be limited because of their transcriptional and regulatory function, respectively [4]. The greater discriminating power of SSRs that are less (IN) or equally (UP) polymorphic than SSRs in other genomic regions corroborates the hypothesis that evolution of SSRs located in these functional genomic regions is not neutral, but may be directional and under the same type of evolution pressure as the locus they are associated with [2, 37]. In fact, a variation of repeat units in regions upstream of coding genes, i.e. promoter regions, may affect transcription and/or translation, possibly leading to phenotypic effects [4]. This interpretation is also supported by the observation that the pattern of frequency of different types of SSRs is identical in the UP regions and within exons, and clearly different from patterns observed in other genomic regions. It has been previously demonstrated that genetic variation supplied by SSRs could be partially responsible for phenotypic differences both at the interspecific level (as documented in voles, where some social behaviour seems to be affected by SSR length in 5’ of *avpr1a*, a gene encoding for a vasopressin receptor, [38]) and at intraspecific level (as reported in dogs, in which 17 genes influencing morphological traits contain polymorphisms in repetitive elements, [39]). For instance, polymorphisms in SSR repeats within the promoter region of the yeast RAS2 locus, a gene assumed to be involved in modulating sporulation efficiency, could be linked to differences in sporulation efficiency in different strains [2].

## Conclusions

In conclusion, this is one of the first reports linking the presence and type of SSRs with DNA regions upstream of 5’UTR. Although definitive experimental confirmation is needed (e.g., functional genomics assays), this study supports the notion that selection processes occur almost exclusively in favour of trinucleotides in the 5’UTR in fungi, a well-known mechanism in other groups of organisms. It could be inferred that SSRs in different genomic locations may be evolving following different rates and evolutionary trajectories. From an applied perspective, while SSRs away from transcribed regions may be better to identify different genotypes, SSRs within ORFs or immediately upstream 5’UTR may be more powerful to study diverging populations or closely related sibling species.

## Methods

### *In silico* SSRs identification and localisation in *H. irregulare* genome

The available genome of *H. irregulare* ([40]; http://genome.jgi-psf.org/Hetan2/Hetan2.home.html) was searched for SSRs using SciRoKo [41] with the ‘perfect MISA-mode’ search set at a minimum number of repeats of 14 mononucleotides, 7 dinucleotides, 5 trinucleotides, 4 tetranucleotides, 4 pentanucleotides, 4 hexanucleotides. A Python script detecting gene annotations (http://biopython.org/wiki/Intergenic_regions) was customized (Additional file 3) and used to distinguish between intergenic and intragenic regions.

The distribution of SSRs was further described by manual annotation of all microsatellites present in the 10 largest scaffolds, representing 75.33 % of the genome. SSRs were scored as present or absent in the following genomic regions: exons, introns, 3’UTRs and 5’UTRs. Since gene expression is reported to be controlled by promoters located before transcribed regions [42], SSRs present within 50 bp and within 50–500 bp upstream or downstream of UTRs were manually annotated. Finally, all SSRs located further than 500 bp from UTRs were also annotated. Manual annotation was conducted using all JGI filtered gene models of *H. irregulare*, which includes gene models selected manually as the best representative model available for each gene. The frequency of SSRs was standardized based on the percentage of each of the above genomic regions as in the Frozen Catalog 090414. A Chi-square test was used to test the H_0_ hypothesis corresponding to a random distribution of the SSRs in the 10 genomic regions analyzed. SSRs positions inferred by SciRoKo were scanned to identify SSRs located in exons.

In addition, transcripts harbouring SSRs were identified by using SciRoKo on the coding sequences of *H. irregulare* (Hannosum_v2.FilteredModels1.CDS.fasta) and their corresponding encoded proteins were analyzed with Blast2GO [43] to search for homologs and to determine their putative function and their Gene Ontology (GO) annotation. Significant differences in frequencies of GO terms compared to all *H. irregulare* gene models were assessed by using a Fisher's exact test [44] integrated in Blast2GO.

### SSRs polymorphisms analysis

#### Biological materials and DNA extraction

A total of 51 haploid genotypes of *Heterobasidion* spp. isolated from single spores germinated on wood discs exposed in forest were used as samples. The samples, which included 20 isolates of *H. irregulare* and 31 isolates of *H. annosum*, were deposited at Garbelotto Laboratory collection (Additional file 4). All genotypes were collected in Italy and grown for ten days in multi-well culture plates filled with 2 ml of 2 % Malt Extract Agar (MEA) before being harvested. Mycelia were collected in 2 ml tubes by scraping the surface of cultures with a sterile scalpel and lyophilized over night.

DNA extraction was performed on 20 mg lyophilized mycelia and eluted in 50 μl ultra-pure water using the Puregene DNA isolation kit (Gentra System, Inc., Minneapolis, MI, USA) according to the manufacturer’s protocol. For few samples, another method to extract DNA was used consisting of adding 200 μl di 0.5 mM of NaOH [45] on 20 mg of lyophilized mycelia and, after vortexing, 5 μl of solution was added in a 1.5 ml tube containing 495 μl of 100 mM TrisHCl (pH 8.00) and stored in a freezer at −20 °C.

#### Primer design and development of SSR markers

By using data generated by *in silico* analysis, primers were specifically designed on flanking sequences of loci containing trinucleotide SSRs. In detail, 11 primer pairs were designed for OUT loci, 9 primer pairs for UP loci, 13 primer pairs for IN loci, 6 primer pairs for DOWN loci. All primers were designed using Primer3Plus (http://www.bioinformatics.nl/cgi-bin/primer3plus/primer3plus.cgi/) and tested with PrimerBLAST (http://www.ncbi.nlm.nih.gov/tools/primer-blast/) to verify they did not match other genomic regions. Universal fluorescent labelling (UFL) was used for the detection of the polymorphisms of SSR markers [46]. This system is based on PCR driven by forward specific primers and a reporter primer 5′ fluorescent labeled. The forward sequence-specific primer is designed to contain a tag region which act as template for the 5′ labeled reporter primer. The resulting amplicons can be easily analyzed with an automated sequencer [46]. In this work, the forward sequence of each specific primer was designed with a fluorescent labeled tag region (5′-6-carboxyfluorescein (FAM)-GGTGGCGACTCCTGGAG-3′) bearing no homology to any sequence [46]. Primer sequences are reported in Additional file 1.

#### SSRs amplification and polymorphism analysis

Screening and genotyping of *Heterobasidion* genotypes were performed with all the 39 SSRs markers developed. Markers were used with the following PCR reactions, in which the annealing temperature and time were optimized for each primer using a gradient PCR. Each PCR reaction was performed in 0.2 ml tubes with 25 μl of the reaction mixture containing of 1X buffer Green, 0.2 mM dNTPs, 1.5 mM MgCl_2_, 0.5 mg/ml bovine serum albumin (BSA), 0.2 μM of each primer forward, 0.2 μM of each primer reverse, 0.2 μM of florescent FAM dye and 1U of Taq polymerase (Promega, Madison, WI, USA). DNA amplification was performed using an initial denaturation step of 94 °C for 1 min followed by 30 cycles as 94 °C for 30s, annealing temperature 58–60 °C, depending on the primer pair, for 30–45 s and elongation at 72 °C for 30s, followed by a final extension at 72 °C for 5 min. Optimized annealing temperature for each primer pair is reported in Additional file 1. Amplification products were diluted with deionized formamide and GeneScan-Liz500, denaturized for 5 min at 95 °C followed by 5 min at 4 °C. Size analyses were performed by capillarity electrophoresis on an automated ABI 3730 Genetic Analyzer using Peak Scanner version 3.5 (Applied Biosystems). Electropherograms were manually scored to determine the allelic size of each locus analyzed and a matrix including all genetic data was generated.

#### Statistical and population genetics analysis

Genetic data of 20 genotypes of *H. irregulare* from two populations and 31 genotypes of *H. annosum* from three populations, generated by 39 microsatellite markers, were analyzed by using Arlequin v3.5 [47], to determine the fixation index (Fst) among populations based on the number of different alleles. Each multilocus genotype was treated as an haplotype. Significance level of differentiation was estimated by setting 10,000 permutations at *p*-value < 0.05. By using Arlequin v3.5, hierarchical analysis of molecular variance, i.e. AMOVA, was performed to determine the partitioning of genetic variance between the two fungal species, among population within each species, and among genotypes within populations. For each of the 39 loci, Fst was used to calculate the gene flow (2 Nm) according to the formula Fst = 1 / (4 Nm + 1). The average number of alleles for each population was estimated using GenAlEx v6.5 software [48], as well as the number of effective alleles, the number of private and shared alleles *per* species. Principal Coordinate Analysis (PCoA) was performed to define the genetic relationships among detected populations on the basis of the genetic distance matrix created with GenAlEx. The F-test in Past3 [49] was used for testing differences in variance detected by analyzing the molecular variance of markers located in different genomic regions (i.e. OUT, UP, IN and DOWN).

## Availability of supporting data

The data sets supporting the results of this article are included within the article and its additional files.

## Additional files

Additional file 1:
**SSRs markers used to determine the genetic variability of the five populations of** ***H. irregulare*** **and** ***H. annosum*** **.** (DOCX 17 kb) Additional file 2:
**Locus** ***per*** **locus fixation index (Fst) and gene flow (2Nm) among 2 distinct sampling populations of** ***H. irregulare***, **both from Latium, and from 3 populations of** ***H. annosum*** **from various regions of Northern and central Italy, including Latium** **.** (DOCX 14 kb) Additional file 3:
**Python script used to distinguish between intergenic and intragenic regions based on** ***H. irregulare*** **gene annotations** **.** (TXT 2 kb) Additional file 4:
**List of** ***Heterobasidion*** **genotypes analyzed** **.** (DOCX 14 kb)

## References

[1] Haasl RJ, Payseur BA (2013). Microsatellites as targets of natural selection. Mol Biol Evol.

[2] Kashi Y, King DG (2006). Simple sequence repeats as advantageous mutators in evolution. Trends Genet.

[3] Buschiazzo E, Gemmell NJ (2006). The rise, fall and renaissance of microsatellites in eukaryotic genomes. Bioessays.

[4] Li Y-C, Korol AB, Fahima T, Beiles A, Nevo E (2002). Microsatellites: genomic distribution, putative functions and mutational mechanisms: a review. Mol Ecol.

[5] Ivors K, Garbelotto M, Vries IDE, Ruyter-Spira C, Hekkert BT, Rosenzweig N (2006). Microsatellite markers identify three lineages of *Phytophthora ramorum* in US nurseries, yet single lineages in US forest and European nursery populations. Mol Ecol.

[6] Zane L, Bargelloni L, Patarnello T (2002). Strategies for microsatellite isolation: a review. Mol Ecol.

[7] Li Y-C, Korol AB, Fahima T, Nevo E (2004). Microsatellites within genes: structure, function, and evolution. Mol Biol Evol.

[8] Ellegren H (2004). Microsatellites: simple sequences with complex evolution. Nat Rev Genet.

[9] Karaoglu H, Lee CMY, Meyer W (2005). Survey of simple sequence repeats in completed fungal genomes. Mol Biol Evol.

[10] Verstrepen KJ, Jansen A, Lewitter F, Fink GR (2005). Intragenic tandem repeats generate functional variability. Nat Genet.

[11] Wagner GP, Lynch VJ (2008). The gene regulatory logic of transcription factor evolution. Trends Ecol Evol.

[12] Gemayel R, Vinces MD, Legendre M, Verstrepen KJ (2010). Variable tandem repeats accelerate evolution of coding and regulatory sequences. Annu Rev Genet.

[13] Garbelotto M, Gonthier P (2013). Biology, epidemiology, and control of *Heterobasidion* species worldwide. Ann Rev Phyto.

[14] Stenlid J, Karlsson JO (1991). Partial intersterility in *Heterobasidion annosum*. Mycol Res.

[15] Otrosina WJ, Chase TE, Cobb FW, Korhonen K (1993). Population structure of *Heterobasidion annosum* from North America and Europe. Can J Bot.

[16] Linzer RE, Otrosina WJ, Gonthier P, Bruhn J, Laflamme G, Bussières G (2008). Inferences on the phylogeography of the fungal pathogen *Heterobasidion annosum*, including evidence of interspecific horizontal genetic transfer and of human-mediated, long-range dispersal. Mol Phylogenet Evol.

[17] Dalman K, Olson Å, Stenlid J (2010). Evolutionary history of the conifer root rot fungus *Heterobasidion annosum* sensu lato. Mol Ecol.

[18] Sillo F, Garbelotto M, Friedman M, Gonthier P (2015). Comparative genomics of sibling fungal pathogenic taxa identifies adaptive evolution without divergence in pathogenicity genes or genomic structure. Genome Biol Evol.

[19] Gonthier P, Warner R, Nicolotti G, Mazzaglia A, Garbelotto MM (2004). Pathogen introduction as a collateral effect of military activity. Mycol Res.

[20] Gonthier P, Nicolotti G, Linzer R, Guglielmo F, Garbelotto M (2007). Invasion of European pine stands by a North American forest pathogen and its hybridization with a native interfertile taxon. Mol Ecol.

[21] Gonthier P, Garbelotto M (2011). Amplified fragment length polymorphism and sequence analyses reveal massive gene introgression from the European fungal pathogen *Heterobasidion annosum* into its introduced congener *H. irregulare*. Mol Ecol.

[22] Garbelotto M, Guglielmo F, Mascheretti S, Croucher PJP, Gonthier P (2013). Population genetic analyses provide insights on the introduction pathway and spread patterns of the North American forest pathogen *Heterobasidion irregulare* in Italy. Mol Ecol.

[23] Gonthier P, Anselmi N, Capretti P, Bussotti F, Feducci M, Giordano L (2014). An integrated approach to control the introduced forest pathogen *Heterobasidion irregulare* in Europe. Forestry.

[24] Tóth G, Gáspári Z, Jurka J (2000). Microsatellites in different eukaryotic genomes: survey and analysis. Genome Res.

[25] Metzgar D, Bytof J, Wills C (2000). Selection against frameshift mutations limits microsatellite expansion in coding DNA. Genome Res.

[26] Li C-Y, Liu L, Yang J, Li J-B, Su Y, Zhang Y (2009). Genome-wide analysis of microsatellite sequence in seven filamentous fungi. Interdiscip Sci Comput Life Sci.

[27] Dokholyan N, Buldyrev S, Havlin S, Stanley H (2000). Distributions of dimeric tandem repeats in non-coding and coding DNA sequences. J Theor Biol.

[28] Young ET, Sloan JS, Van Riper K (2000). Trinucleotide repeats are clustered in regulatory genes in *Saccharomyces cerevisiae*. Genetics.

[29] Richards RI, Holman K, Yu S, Sutherland GR (1993). Fragile X syndrome unstable element, p(CCG)n, and other simple tandem repeat sequences are binding sites for specific nuclear proteins. Hum Mol Genet.

[30] Karlin S, Campbell AM, Mrázek J (1998). Comparative DNA analysis across diverse genomes. Annu Rev Genet.

[31] Stallings RL (1994). Distribution of trinucleotide microsatellites in different categories of mammalian genomic sequence: implications for human genetic diseases. Genomics.

[32] Murat C, Riccioni C, Belfiori B, Cichocki N, Labbé J, Morin E (2011). Distribution and localization of microsatellites in the Perigord black truffle genome and identification of new molecular markers. Fungal Genet Biol.

[33] Labbé J, Murat C, Morin E, Tacon FL, Martin F (2010). Survey and analysis of simple sequence repeats in the *Laccaria bicolor* genome, with development of microsatellite markers. Curr Genet.

[34] Mun J-H, Kim D-J, Choi H-K, Gish J, Debellé F, Mudge J (2006). Distribution of microsatellites in the genome of *Medicago truncatula*: a resource of genetic markers that integrate genetic and physical maps. Genetics.

[35] Lawson MJ, Zhang L (2006). Distinct patterns of SSR distribution in the *Arabidopsis thaliana* and rice genomes. Genome Biol.

[36] Qian J, Xu H, Song J, Xu J, Zhu Y, Chen S (2013). Genome-wide analysis of simple sequence repeats in the model medicinal mushroom *Ganoderma lucidum*. Gene.

[37] Lim S, Notley-McRobb L, Lim M, Carter DA (2004). A comparison of the nature and abundance of microsatellites in 14 fungal genomes. Fungal Genet Biol.

[38] Hammock EAD, Young LJ (2004). Functional microsatellite polymorphism associated with divergent social structure in vole species. Mol Biol Evol.

[39] Fondon JW, Garner HR (2004). Molecular origins of rapid and continuous morphological evolution. Proc Natl Acad Sci U S A.

[40] Olson A, Aerts A, Asiegbu F, Belbahri L, Bouzid O, Broberg A (2012). Insight into trade-off between wood decay and parasitism from the genome of a fungal forest pathogen. New Phytol.

[41] Kofler R, Schlötterer C, Lelley T (2007). SciRoKo: a new tool for whole genome microsatellite search and investigation. Bioinformatics.

[42] Abeel T, Saeys Y, Bonnet E, Rouzé P, Peer YV (2008). Generic eukaryotic core promoter prediction using structural features of DNA. Genome Res.

[43] Conesa A, Götz S, García-Gómez JM, Terol J, Talón M, Robles M (2005). Blast2GO: a universal tool for annotation, visualization and analysis in functional genomics research. Bioinformatics.

[44] Bluthgen N, Brand K, Cajavec B, Swat M, Herzel H, Beule D (2005). Biological profiling of gene groups utilizing Gene Ontology. Genome Inform.

[45] Osmundson TW, Eyre CA, Hayden KM, Dhillon J, Garbelotto MM (2013). Back to basics: an evaluation of NaOH and alternative rapid DNA extraction protocols for DNA barcoding, genotyping, and disease diagnostics from fungal and oomycete samples. Mol Ecol Resour.

[46] Shimizu M, Kosaka N, Shimada T, Nagahata T, Iwasaki H, Nagai H (2002). Universal Fluorescent Labeling (UFL) method for automated microsatellite analysis. DNA Res.

[47] Excoffier L, Laval G, Schneider S (2007). Arlequin (version 3.0). An integrated software package for population genetics data analysis. Evol Bioinform Online.

[48] Peakall R, Smouse PE (2006). genalex 6: Genetic analysis in Excel. Population genetic software for teaching and research. Mol Ecol Notes.

[49] Hammer Ø, Harper DAT, Ryan PD (2001). PAST: paleontological statistics software package for education and data analysis. Palaeontol Electron.

