# Supplementary material for: Selection processes in simple sequence repeats suggest a correlation with their genomic location: insights from a fungal model system
Source: BMC Genomics. 2015 Dec 29;16:1107. doi: 10.1186/s12864-015-2274-x (PMC4696308; doi:10.1186/s12864-015-2274-x)
Supplement: Additional file 1: — SSRs markers used to determine the genetic variability of the five populations of H. irregulare and H. annosum . (DOCX 17 kb) [file 12864_2015_2274_MOESM1_ESM.docx]

**Additional file 1.** SSRs markers used to determine the genetic variability of the five populations of *H. irregulare* and *H. annosum*.

| **Position** | **SSR name** | **Forward primer (5'-3')** | **Reverse primer (3'-5')** | **Ta (°C)** | **Repeat motif** | **Allele size range (bp)** | **N° repeats** |
| --- | --- | --- | --- | --- | --- | --- | --- |
| Loci OUT | MS01_s1_out | CTGGTCTGAGGGAAGTGCAG | TACCAGCACCTCCTCGTACA | 60 | CAG | 188-196 | 9-13 |
|  | MS02_s1_out | CGACCTGGAGAGACGTAAAGC | GGCAGAGAGGGAGGTATGGA | 60 | CCT | 164-217 | 2-19 |
|  | MS03_s1_out | CGTTGAGGGAGGAGGACATG | CTCCCAACACCGTCCTGATC | 59.5 | GAA | 141-156 | 5-10 |
|  | MS15_s2_out | GTGTGAGCATGAACGTTGGA | AACACTGGCCTCACTGATGT | 60 | GCA | 198-215 | 7-12 |
|  | MS26_s3_out | AAGATGAGAACGACGCGGAT | CTGTGTCTTCGGCTGTTTCG | 58 | AGA | 132-155 | 9-16 |
|  | MS38_s5_out | CCGGACTTGAGTTTGAACGT | GCCTGTTGTGAATCCTTGCA | 60 | CAT | 213-389 | 7-68 |
|  | MS57_s7_out | CTGTTGCTGCTGTAGTTGTTGT | CGAAGAAGGAGCAGGTGGAG | 60 | TGC | 177-248 | 13-37 |
|  | MS61_s8_out | GGGAGGTACATCGCCGAC | CATCAGCACTCGTCCCCG | 60 | TGC | 165-217 | 5-22 |
|  | MS63_s8_out | ACAGCCTTTCTCCACACTCG | ACAGCCTTTCTCCACACTCG | 60 | TCG | 208-217 | 14-17 |
|  | MS72_s10_out | GATTGTCTTTGGCGTCGCAG | GGCGAAGAAGAGGAAGGAGG | 60 | CGA | 158-256 | 8-13 |
|  | MS75_s11_out | CAACAGCCTCCTCCAGCC | GGTCGTGGCGGTGTGTATC | 59 | CCA | 127-475 | 4-112 |
| Loci UP | MS08_s1_up | TACACGTCATGGGCACTCTC | CCTCTCTCCCTCGTTCCTCA | 58 | TGC | 155-184 | 3-14 |
|  | MS09_s1_up | GGTGCTTGGAGAGGGACATG | GTCTACGCCGCCATCCAC | 60 | GAG | 180-213 | 3-15 |
|  | MS17_s2_up | CTGCTCTTCCATGTCTTCGC | GAGGAAGTCGTAGCGTGAGG | 60 | TCG | 140-210 | 2-25 |
|  | MS18_s2_up | TGGTCGTGCTGTGATTTACG | CCTCAGGCTGCTATCGAAATC | 60 | ATG | 82-122 | 3-17 |
|  | MS28_s3_up | GCCTAGATTGCCGAACATGG | ACGTGTTTAGCGTTTGGTCC | 60 | GCT | 111-149 | 3-16 |
|  | MS29_s3_up | CATGAATAGAGGAGCAGCGG | ACCCTCTAGTGCATGTCCAG | 59 | GTG | 210-236 | 15-28 |
|  | MS40_s5_up | TAGGGACACTTGTAAGGCCG | AACCATCACCATACCACCGT | 60 | GGC | 172-325 | 7-58 |
|  | MS65_s8_up | GGAGATGAAAGAGCGGTGGA | CTGGACGTTCTTGGCTGACT | 60 | GAC | 160-172 | 11-15 |
|  | MS66_s8_up | GTGACTGCGTGACGAGAGAG | CGGCGACAATGAGACAGGAT | 60 | CTC | 193-217 | 7-15 |
| Loci IN (Inside ORFs) | MS04_s1_in | TCCTCGTCGTCATCATCACT | AGCGGAAAAAGAGGAGGAAG | 60 | GAC | 102-204 | 6-40 |
|  | MS06_s1_in | CTGCTGGGAGGTGTTCATC | GATCCCGTTCACGTTCGAC | 60 | CGG | 103-133 | 9-27 |
|  | MS12_s1_in | CGTCGCTCGAGACGTAGAC | CTACAGCCGCTTCCTTCG | 60 | CAG | 105-137 | 6-17 |
|  | MS13_s1_in | GGAGGCCCTTGAGGAAGC | CGCGAGATCGGTGGAGAT | 58.5 | ATC | 110-183 | 9-31 |
|  | MS19_s2_in | GAGCCGCACCCTCATCAC | AACAACATCACGTTCGACCA | 60 | CCA | 102-674 | 1-191 |
|  | MS20_s2_in | GCTCTTCCCGCGCTAACT | GCTTCGATCTCAACAGCAATC | 60 | GAC | 109-123 | 11-13 |
|  | MS27_s2_in | CAGAAAGCGAAAACAGAGGAG | GACGACGAACTCGTGGAAG | 60 | CTC | 112-156 | 8-20 |
|  | MS34_s3_in | GAGGAGGTCGGAGGAGGAC | CTCGGAAGACTCTGGCGTA | 60 | ACC | 102-118 | 7-13 |
|  | MS35_s3_in | GTGTGGTTCGGTTGTTCTGG | CACCTGTCTCGCCCTTCC | 58.5 | GCT | 103-158 | 6-25 |
|  | MS36_s3_in | ATCGAGTCGCCTCTCTGC | GCTCGACCAATCCGAATC | 60 | CGT | 124-187 | 6-11 |
|  | MS43_s4_in | TGCTATAGGGCAAAGATGCTG | CATTGAAGGCGTCAACCAG | 60 | AGC | 111-195 | 7-53 |
|  | MS70_s7_in | GGATTTCGATGCCCCAAG | AGATGGGGATGAGGGACATT | 60 | GAG | 129-523 | 10-30 |
|  | MS74_s7_in | GGATCCGACTTATCCACGTC | GAAGACGTGCCTGACAGTGA | 60 | GAC | 108-126 | 7-11 |
| Loci DOWN | MS22_s2_down | TGGAGAATTAGGAGTGGGCG | ATGAACACTGGCCTCACTGA | 59 | GCA | 112-214 | 8-43 |
|  | MS32_s3_down | AGTCATGGGAGAACGAGTGG | GAAGGAAAGGTACGTCGCA | 60 | ACG | 158-304 | 0-47 |
|  | MS33_s3_down | CCACCCTCCTCTTCATCCAA | ACGACGTTCATCCTCAACCT | 59.5 | TGG | 115-189 | 9-33 |
|  | MS34_s3_down | TTGAACGTACTGGTCGCAAG | GAAGTTGTGGCCTGCAAAGA | 60 | GGC | 192-206 | 17-21 |
|  | MS69_s8_down | CCCTGACCCTGAACATGTCC | ATGGGCATGCGATACCTGC | 60 | CTG | 146-216 | 5-28 |
|  | MS77_s11_down | TACTTACCTACCGTCGCCGT | CTCCCGCCGTTTGTTTGTTT | 60 | GAT | 170-223 | 2-21 |
