# Supplementary material for: Selection processes in simple sequence repeats suggest a correlation with their genomic location: insights from a fungal model system
Source: BMC Genomics. 2015 Dec 29;16:1107. doi: 10.1186/s12864-015-2274-x (PMC4696308; doi:10.1186/s12864-015-2274-x)
Supplement: Additional file 2: — Locus per locus fixation index (Fst) and gene flow (2Nm) among 2 distinct sampling populations of H. irregulare, both from Latium, and from 3 populations of H. annosum from various regions of Northern and central Italy, including Latium . (DOCX 14 kb) [file 12864_2015_2274_MOESM2_ESM.docx]

**Additional file 2.** Locus *per* locus fixation index (Fst) and gene flow (Nm) among 2 distinct sampling populations of *H. irregulare*, both from Latium, and from 3 populations of *H. annosum* from various regions of Northern and central Italy, including Latium.

| **Loci** | **Fst** | | **2Nm** | **AVERAGE 2Nm** |
| --- | --- | --- | --- | --- |
| **OUT** | |  |  |  |
| MS01_s1_out | 0.30232 | | 1.154 | 1.181 |
| MS02_s1_out | 0.33683 | | 0.984 |  |
| MS03_s1_out | 0.44197 | | 0.631 |  |
| MS15_s2_out | 0.71181 | | 0.202 |  |
| MS26_s3_out | 0.5231 | | 0.456 |  |
| MS38_s5_out | 0.1569 | | 2.687 |  |
| MS57_s7_out | 0.27549 | | 1.315 |  |
| MS61_s8_out | 0.17186 | | 2.409 |  |
| MS63_s8_out | 0.20772 | | 1.907 |  |
| MS72_s10_out | 0.37213 | | 0.844 |  |
| MS75_s11_out | 0.55709 | | 0.398 |  |
| **UP** | |  |  |  |
| MS08_s1_up | 0.25319 | | 1.475 | 0.880 |
| MS09_s1_up | 0.42637 | | 0.673 |  |
| MS17_s2_up | 0.4265 | | 0.672 |  |
| MS18_s2_up | 0.29672 | | 1.185 |  |
| MS28_s3_up | 0.733 | | 0.182 |  |
| MS29_s3_up | 0.37856 | | 0.821 |  |
| MS40_s5_up | 0.20944 | | 1.887 |  |
| MS65_s8_up | 0.64422 | | 0.276 |  |
| MS66_s8_up | 0.40162 | | 0.745 |  |
| **IN** | |  |  |  |
| MS04_s1_in | 0.18778 | | 2.163 | 1.400 |
| MS06_s1_in | 0.21281 | | 1.850 |  |
| MS12_s1_in | 0.41767 | | 0.697 |  |
| MS13_s1_in | 0.08842 | | 5.155 |  |
| MS19_s2_in | 0.12748 | | 3.422 |  |
| MS20_s2_in | 0.88337 | | 0.066 |  |
| MS27_s2_in | 0.35876 | | 0.894 |  |
| MS34_s3_in | 0.70325 | | 0.211 |  |
| MS35_s3_in | 0.20738 | | 1.911 |  |
| MS36_s3_in | 0.89441 | | 0.059 |  |
| MS43_s4_in | 0.27535 | | 1.316 |  |
| MS70_s7_in | 0.53899 | | 0.428 |  |
| MS74_s7_in | 0.94459 | | 0.029 |  |
| **DOWN** | |  |  |  |
| MS22_s2_down | 0.3153 | | 1.086 | 1.532 |
| MS32_s3_down | 0.38322 | | 0.805 |  |
| MS33_s3_down | 0.48413 | | 0.533 |  |
| MS34_s3_down | 0.14789 | | 2.881 |  |
| MS69_s8_down | 0.15086 | | 2.814 |  |
| MS77_s11_down | 0.3181 | | 1.072 |  |
