# Supplementary material for: Selection processes in simple sequence repeats suggest a correlation with their genomic location: insights from a fungal model system
Source: BMC Genomics. 2015 Dec 29;16:1107. doi: 10.1186/s12864-015-2274-x (PMC4696308; doi:10.1186/s12864-015-2274-x)
Supplement: Additional file 4: — List of Heterobasidion genotypes analyzed . (DOCX 14 kb) [file 12864_2015_2274_MOESM4_ESM.docx]

**Additional file 4.** *Heterobasidion* genotypes analyzed.

| **ID Code** | **Geographic origin** | **species** | **Population Code** |
| --- | --- | --- | --- |
| BM 38 Ec | Ferrara (Italy) | *H. annosum* | EMR-AN |
| BM 40-2a | Ferrara (Italy) | *H. annosum* | EMR-AN |
| BM 41 Nb | Ferrara (Italy) | *H. annosum* | EMR-AN |
| BM 41 Nc | Ferrara (Italy) | *H. annosum* | EMR-AN |
| BM 42 Nd | Ferrara (Italy) | *H. annosum* | EMR-AN |
| BM 42 Ng | Ferrara (Italy) | *H. annosum* | EMR-AN |
| BM 42 Sa | Ferrara (Italy) | *H. annosum* | EMR-AN |
| BM 42 Se | Ferrara (Italy) | *H. annosum* | EMR-AN |
| BM 43 Sa | Ferrara (Italy) | *H. annosum* | EMR-AN |
| BM 43 Sb | Ferrara (Italy) | *H. annosum* | EMR-AN |
| 5EH | Castelfusano (Rome, Italy) | *H. irregulare* | LZO5-IR |
| 8OB | Castelfusano (Rome, Italy) | *H. irregulare* | LZO5-IR |
| 9OA | Castelfusano (Rome, Italy) | *H. irregulare* | LZO5-IR |
| 11NA | Castelfusano (Rome, Italy) | *H. irregulare* | LZO5-IR |
| 39NE | Castelfusano (Rome, Italy) | *H. irregulare* | LZO5-IR |
| 58OA | Anzio (Rome, Italy) | *H. irregulare* | LZO5-IR |
| 87NB | Nettuno (Rome, Italy) | *H. irregulare* | LZO5-IR |
| 87OA | Nettuno (Rome, Italy) | *H. irregulare* | LZO5-IR |
| 89EG | Nettuno (Rome, Italy) | *H. irregulare* | LZO5-IR |
| 89NA | Nettuno (Rome, Italy) | *H. irregulare* | LZO5-IR |
| 89OB | Nettuno (Rome, Italy) | *H. irregulare* | LZO5-IR |
| 90SC | Nettuno (Rome, Italy) | *H. irregulare* | LZO5-IR |
| 91NA | Nettuno (Rome, Italy) | *H. irregulare* | LZO5-IR |
| 50EA | Circeo Nettuno (Rome, Italy) | *H. irregulare* | LZO6-IR |
| 115NA | Circeo North area (Rome, Italy) | *H. irregulare* | LZO6-IR |
| 118ND | Circeo North area (Rome, Italy) | *H. irregulare* | LZO6-IR |
| 127NA | Circeo North area (Rome, Italy) | *H. irregulare* | LZO6-IR |
| 132NF | Circeo North area (Rome, Italy) | *H. irregulare* | LZO6-IR |
| 135ED | Circeo Nettuno (Rome, Italy) | *H. irregulare* | LZO6-IR |
| 12SA | Castelfusano Nettuno (Rome, Italy) | *H. irregulare* | LZO-AN |
| 41OC | Circeo National Park Sabaudia Nettuno (Rome, Italy) | *H. annosum* | LZO-AN |
| 46SF | Circeo National Park Sabaudia Nettuno (Rome, Italy) | *H. annosum* | LZO-AN |
| 58EA | Gallinara Park (Rome, Italy) | *H. annosum* | LZO-AN |
| 137OB | Circeo National Park Sabaudia (Rome, Italy) | *H. annosum* | LZO-AN |
| 139OE | Circeo National Park Sabaudia (Rome, Italy) | *H. annosum* | LZO-AN |
| 142EA | Circeo National Park Sabaudia (Rome, Italy) | *H. annosum* | LZO-AN |
| 142NA | Circeo National Park Sabaudia (Rome, Italy) | *H. annosum* | LZO-AN |
| 143OA | Circeo National Park Sabaudia (Rome, Italy) | *H. annosum* | LZO-AN |
| 145SB | Circeo National Park Sabaudia (Rome, Italy) | *H. annosum* | LZO-AN |
| 3WB | Val Sesia (Vercelli, Italy) | *H. annosum* | PVA-AN |
| 3.3 | Trasquera (Verbania, Italy) | *H. annosum* | PVA-AN |
| 3.6 | Trasquera (Verbania, Italy) | *H. annosum* | PVA-AN |
| 4EC | Val Sesia (Vercelli, Italy) | *H. annosum* | PVA-AN |
| 5A | Gressan Chanté (Aosta, Italy) | *H. annosum* | PVA-AN |
| 7C | Toceno (Verbania, Italy) | *H. annosum* | PVA-AN |
| 8X | Aymavilles (Aosta, Italy) | *H. annosum* | PVA-AN |
| 9N | Aymavilles (Aosta, Italy) | *H. annosum* | PVA-AN |
| 11WD | Val Sesia (Vercelli, Italy) | *H. annosum* | PVA-AN |
| 12NG | Val Sesia (Vercelli, Italy) | *H. annosum* | PVA-AN |
| 16SA | Val Sesia (Vercelli, Italy) | *H. annosum* | PVA-AN |
| 19SE | Val Sesia (Vercelli, Italy) | *H. annosum* | PVA-AN |
|  |  |  |  |
